# Efficacy of periodontal treatment modalities in Down syndrome patients: a systematic review and meta-analysis

**DOI:** 10.1038/s41432-024-01055-x

**Published:** 2024-08-25

**Authors:** Zakaria Yehia, Angelika Silbereisen, Despina Koletsi, Mahla Arabzadehtousi, Georgios Tsilingaridis, Nagihan Bostanci

**Affiliations:** 1 Division of Periodontology and Oral Health, Department of Dental Medicine, Karolinska Institutet, Stockholm, Sweden; 2 Department of Orthodontics, Folktandvården Stockholms län AB, Folktandvården Eastmaninstitutet, Stockholm, Sweden; 3 Clinic of Orthodontics and Pediatric Dentistry, Center of Dental Medicine, University of Zurich, Zurich, Switzerland; 4 Meta-Research Innovation Center at Stanford (METRICS), Stanford University, Stanford, CA USA; 5 Division of Orthodontics and Pediatric Dentistry, Department of Dental Medicine, Karolinska Institutet, Stockholm, Sweden; 6 Centre of Pediatric Oral Health, Stockholm, Sweden

**Keywords:** Dental diseases, Dental caries, Periodontitis

## Abstract

**Objective:**

The systematic review aimed to review the existing evidence, to identify and appraise the effectiveness of periodontal prevention and treatment modalities in individuals diagnosed with Down syndrome (DS) and to determine the estimates of the effects of implemented periodontal prevention and treatment strategies compared to chromosomally normal (CN) individuals.

**Methodology:**

The systematic review was conducted and reported in conformity with the PRISMA (Preferred Reporting Items for Systematic Reviews and Meta-Analysis) guidelines. The study protocol was registered in the Open Science Framework. Electronic and manual searches, in accordance with PICO framework and delineated inclusion/exclusion criteria, were conducted in multiple databases.

**Results:**

The initial search identified 11,704 studies. After removing duplicates, 9,048 remained. Title and abstract screening narrowed these to 281 for full-text review. Ultimately, 16 studies met the inclusion criteria, with 4 eligible for quantitative data synthesis. Results of the meta-analysis indicated that professional tooth cleaning in combination with oral hygiene reinforcement was less effective in the reduction of PPD in patients with DS compared to those without DS (Mean difference (MD): 0.23; 95% Confidence Interval (CI): 0.14 to 0.32; *p* < 0.001).

**Discussion:**

These findings suggest that conventional periodontal treatment is less effective in managing periodontitis in patients with DS. Thus, tailored periodontal care strategies that address the specific needs of individuals with DS should be implemented to improve treatment outcomes for this population The presence of moderate to high risk of bias in the included studies underscores the need for rigorously designed research that minimizes bias through effective blinding, randomization, control of confounding factors, and inclusion of diverse treatment outcomes to further investigate these associations.

**Conclusion:**

Based on the best available evidence, professional tooth cleaning combined with oral hygiene instructions appears to be less effective in reducing pocket depths in individuals with DS compared to those without DS. 10.17605/OSF.IO/UXTCG

Key points

- Based on the best available evidence, professional tooth cleaning combined with oral hygiene instructions appears to be less effective in reducing probing pocket depths in individuals with Down syndrome (DS) compared to those without DS.
- The current evidence highlights the vulnerability of the DS population to periodontal diseases and emphasizes the need for increased awareness, enhanced monitoring, preventive measures, and personalized treatment planning.
- This systematic review provides new insights into the effectiveness of previously implemented interventions, which may encourage the development of new oral health strategies beneficial not only to individuals with DS but also to those with other abnormalities and intellectual impairments.

## Introduction

Down syndrome (DS) comprises one of the most frequently existing neurodevelopmental birth defects, constituting a chromosomal deviation that is correlating with trisomy of chromosome 21 and with a frequency of 1 per 700–1200 nascencies a year^1,2^. Depending on the type of genetic manifestation, the neurodevelopmental aberration is divided into three types of which primary nondisjunction trisomy 21 measures up to approximately 95 percent of the deviation^3,4^. Mosaicism and translocations, ordinarily of Robertsonian type, accounts for the remaining 5 percent of the aberration^5,6^.

Individuals with DS display phenotypical, physiological, neurological and orofacial manifestations that deviate, alter in severity and considerably impacts the influenced individuals well-being^7^. The clinical manifestation of periodontal disease in these patients is advanced, generalized, and demonstrates a haste progression with an increased disease prevalence of up to 100 percent below the age of 30 years^8,9^. Nevertheless, the progression and severity of periodontal diseases have been demonstrated to be of slower character in terms of alveolar bone loss progression in radiographs over a seven-year period in comparison with previously delineated findings^10,11^. Even though individuals with DS exhibits a severe clinical manifestation of periodontal tissue breakdown, the seriousness goes beyond the breakdown of the periodontal tissues solely caused by confined oral hygiene measures. Greater propensities to inflammatory diseases and infections, notably periodontal infections, may be correlated with immunological aberrations^11–13^. Increased concentrations of Type 1 T helper (Th) 1 and Th2 and Th17-interrelated cytokines have been shown in the gingival crevicular fluid (GCF) in comparison to controls, indicating increased local inflammatory responses and heightened immunological activity. Alterations in the correlation between Th1, Th2 cytokines and interleukin (IL)-4 have as well been exhibited^14^. Moreover, elevated concentrations of matrix metalloproteinase (MMP)2, MMP3, MMP8, MMP9 and fluctuated correlations between MMP8 and Tissue inhibitor of metalloproteinase (TIMP) have been indicated^15^. Orofacial and dental aberrations, saliva properties in terms of salivary electrolyte levels, pH values, flow rates and buffering capacities, and periodontopathogens in the composition of the subgingival microbiota may also account for the periodontal status^16^.

Individuals with DS have been reported to acquire dental health care to a smaller degree in comparison to the general population^17–19^. Caregivers have reported predicaments in executing oral care at home and within the dental clinical settings^19,20^. The significance of a timely introduction to the dental clinical settings and frequent dental recalls have been suggested in order to facilitate consecutive preventive interventions, disease diagnosis and individuals directives in terms of oral hygiene regimens for periodontal disease prevention^17^. In an attempt to assess the effectiveness of preventive programs and treatment modalities for periodontal diseases in individuals with DS, a systematic review concluded that frequent application of chemical adjuvants in combination with periodontal treatment enhances favorable periodontal outcomes regardless of implemented periodontal treatment. Unanimity regarding the use of chlorhexidine was also reported^8^.

Given the suboptimal outcomes and the increased sensitivity for this vulnerable population group on one hand and the paucity of evidence based on systematic reviews (SRs) with meta-analysis in periodontal prevention and treatment strategies, further research is deemed necessary to aid dental practitioners to acquire additional knowledge in oral health intervention strategies. Up to date, no systematic review with meta-analysis has been conducted determining the treatment efficacy of periodontal prevention programs or treatment modalities that would facilitate improvement of oral health in this field and quantitative evidence is limited to epidemiological SRs assessing the association of DS to periodontal disease. Therefore, the aim of this study was to perform a systematic review of the existing evidence base, to identify and appraise the effectiveness of periodontal prevention and treatment modalities in individuals diagnosed with DS and to determine the estimates of the effects of implemented periodontal prevention and treatment strategies compared to chromosomally normal (CN) individuals.

## Methods

This systematic review was conducted and reported in conformity with the Cochrane Handbook of Systematic Reviews of Interventions^21^ and followed the Prefered Reporting Items for Systematic Reviews and Meta-Analysis (PRISMA) guidelines^22^. The study protocol was registered in the Open Science Framework [https://osf.io/uxtcg/].

### Eligibility criteria

The focused research questions was in accordance with the PICO framework^23^ and was delineated as follows: Population: Children, adolescents and adults diagnosed with DS. Interventions: Including but not confined to periodontal prophylaxis, non-surgical periodontal therapy (NSPT) and flap-debridement. Comparisons: Interventions other than those described in the intervention group, or individuals without DS, for example other intellectual impairments or systematically healthy individuals, receiving similar periodontal interventions. Outcome: Any periodontal outcome measure indicating periodontal disease after implementation of interventions. The following delineated inclusion criteria had to be met in order to be eligible and included in the study: Randomized controlled trials, controlled clinical trials, prospective and retrospective cohort studies, case control studies, studies reporting equivalent examinations and comparisons to control groups, studies reporting no less than one quantitative measure of periodontal health status. The exclusion criteria comprised systematic reviews with or without meta-analysis, narrative literature reviews, studies in which periodontal prevention and treatment regimens were not defined, studies without a DS population, experimental studies without control groups, non-clinical studies, case reports, expert opinions and studies in other languages than English.

### Outcome measures and characteristics

The periodontal outcome measures indicating periodontal disease constituted periodontal probing depths (PPD) and reductions in PPD, clinical attachment levels (CAL) and gain in CAL, changes in bleeding indices or bleeding on probing (BoP) scores, changes in plaque indices (PI) or plaque scores, radiographic bone defect changes, changes in the expression of salivary or gingival crevicular fluid (GCF) volumes and markers, patient-reported outcomes including adverse events and adverse reactions.

### Search strategy

In order to identify and retrieve eligible studies, the following databases were electronically searched on the 15th of November 2023: PubMed, EMBASE, Medline via Ovid, Web of Science and Cochrane Central Register of Controlled Trials. The registry for clinical trials ClinicalTrials.gov was searched to identify any ongoing studies. Open Grey was researched in order to distinguish theses, dissertations and unpublished studies. Reference lists was hand searched for any additional inclusion of articles. Combinations of medical subject headings, Emtree subject headings, entry free terms and Boolean operators were utilized in respective database (Supplementary Appendix 1). One reviewer (ZY) conducted and completed the article selection in accordance with delineated inclusion and exclusion criteria. A second reviewer (DK) re-assessed the article selection strategy and was further consulted when uncertainties arose on whether to include or exclude articles. The reviewers were blinded, and the assessments were conducted in parallel. Discrepancies were resolved through an established consensus between reviewers. A third reviewer was consulted if consensus was not reached between reviewers.

### Data extraction and validity assessment

Extraction of data was conducted by one reviewer (ZY) and re-examined by 2 other reviewers (DK, NB) for validation. Discrepancies were discussed among reviewers until a consensus was established. In the case of equivocal or insufficient data sets, authors accountable for the conducted studies were contacted for further data requests and clarifications. Data was documented in standardized piloted forms and included data about study characteristics in terms of study ID, citation, contact details to the authors, study design and type, characteristics of participants, periodontal parameters utilized for diagnosing periodontal disease, implemented periodontal prevention and treatment modalities, outcome data, comparator groups, timeframes for evaluation and information concerning biases.

### Methodological quality assessment and risk of bias

The quality assessment and risk of bias of included studies was conducted by one reviewer (ZY) and validated by 2 other reviewers (DK, NB). The Risk of Bias In Non-Randomized studies – of Interventions (ROBINS – I) assessment tool was utilized for the appraisal of non-randomized studies^24^. For the risk of bias assessment of randomized trials, the Revised Cochrane risk-of-bias tool for randomized trials (RoB 2) was utilized^25^.

### Quantitative syntheses

Following assessment of heterogeneity and considering variability in treatment procedures, populations and settings across studies, random effects meta-analysis were conducted, as applicable. Pooled estimates were presented as mean differences in view of the nature of the pre-defined outcomes, followed by measures of precision (95% confidence intervals, Cls). Statistical heterogeneity was further explored through an I-squared test, where a *p*-value below the level of 10 (i.e, *p* < 0.10) is indicative for non-homogeneity. Visual inspection of heterogeneity as also was based on CI overlap within the constructed forest-plots. An estimate of between study variance was given as tau-squared (τ^2^). In the case of missing data, authors of original studies were contacted for additional data requests. For split-mouth designs, in view of the decreased data variability between quadrants, the following formula was used for the approximation of the standard deviation of the difference: ${{SD}}_{{diff}}=\,\sqrt{{{sd}}_{1}^{2}+{{sd}}_{2}^{2}-2r{{sd}}_{1}{{sd}}_{2}}$ formula where ”r”, the correlation coefficient was set as 0.5 for split-mouth designs (r = 0, for parallel designs)^26^. Publication bias was planned to be explored through standard funnel plots and Egger’s regression test, conditional on the inclusion of more than 10 studies in the quantitative syntheses^27^.

## Results

### Study characteristics

**Fig. 1:**
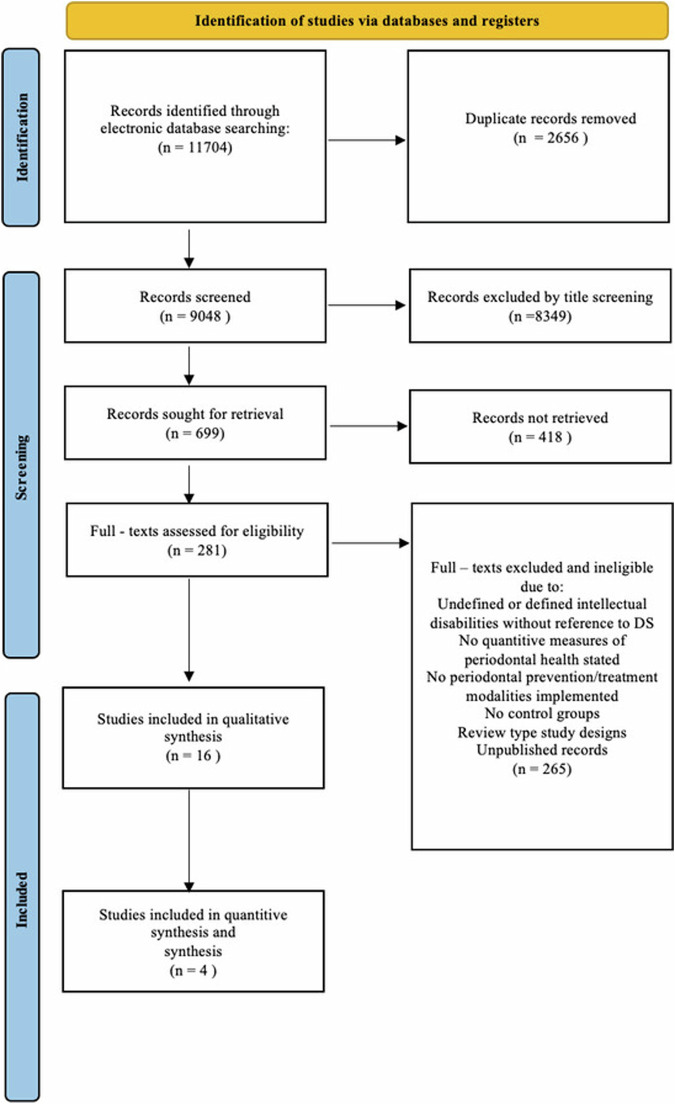
PRISMA flow diagram of study selection process. The figure display the number of records identified through electronic database searching, numbers of records screened, numbers of records excluded in the title, abstract and full-text screenings and the number of records included in the qualitative and quantitative synthesis.

The electronic and manual search resulted in 11,704 studies and ultimately concluded in 9048 studies after the removal of duplicates. The screening of titles and abstracts eventuated in 281 eligible studies for full-text screening. The full-text screening of the selected studies concluded in a final list of 16 included studies that fulfilled the inclusion criteria, of which 4 studies were eligible for quantitative data synthesis for meta-analysis (Fig. 1). Among the 16 included studies, eight constituted randomized clinical trials including two with cross-over designs and two with split-mouth designs^28–35^, three clinical trials including one with cross-over design^12,36,37^, two retrospective cohort studies^38,39^, two prospective longitudinal studies including one with split-mouth design^40,41^ and one with prospective case control study^42^. The included studies were conducted in 11 various countries, five in Brazil, two in Israel, one in Ireland, one in USA, one in Mexico, one in Japan, one in Italy, one in Russia, one in Germany, one in Saudi Arabia and one in Syria. Three studies had institutionalized DS individuals as subjects^31,37,40^.

### Characteristics of subjects

Among the sixteen studies included, the sample sizes of subjects ranged from 8 through 102 participants with ages ranging from 6 to 53 years of age. Regarding gender distribution of subjects, a similar gender allocation could be observed. 192 constituted male subjects and 191 were composed of females of 548 reported participants in total, exhibiting a minor percentage disparity of 2 percent (35 vs. 33 percent). Four studies did not specify the gender distribution^33,36,37,40^. Three studies did not clearly define inclusion and exclusion criteria for recruiting subjects for the studies^31,36,40^. Clinical heterogeneities were observed concerning periodontal disease definitions among the studies. Criterion utilized for defining periodontal disease across included studies constituted: PPD 4 mm, bone loss of 5 mm on radiographs, CAL 5 mm in at least one site, bone loss of 3 mm on radiographs in at least one site, more than eight sites in non-adjacent teeth with PPD 4 mm, CAL 3 mm and BoP, PPD 5 mm, interdental CAL at 2 non-adjacent teeth or buccal or CAL 3 mm with PPD > 3 mm detectable at 2 teeth. Eleven studies did not clearly state a definition for diagnosis periodontal disease^12,28,30–33,35–37,40,41^.

### Periodontal prevention and treatment modalities implemented

Implemented periodontal prevention and treatment modalities of included studies are distinguished in Supplementary Table 1. Eight studies investigated periodontal parameters prior the implementation of prevention or non-surgical treatment modalities and evaluated the outcomes subsequently after^12,33–36,38,39,42^. Two studies evaluated the treatment efficacy of antimicrobial photodynamic therapy as an adjuvant to conventional scaling and root planing compared to conventional scaling and root planing solely^29,34^. Surgical periodontal therapy in the form of open flap debridement was estimated to conventional scaling and root planing in one study^41^. Diverse configurations of chlorhexidine, their treatment efficacies and outcomes on the periodontal status of DS individuals were evaluated in four studies^28,32,37,40^. Three studies compared the effectiveness of electronic and manual toothbrushes on the periodontal status^30,33,35^. One study evaluated the treatment efficacy of periodic topical Kanamycin on the periodontal status^31^. In addition to patient-centered parameters, the perception and involvement of parents, guardians and caregivers were evaluated in all studies except one^40^.

### Periodontal outcomes

The primary focus was on mean PD while other periodontal outcomes included mean CAL, mean changes in BoP and bleeding indices, mean changes in PI and plaque indices/scores, mean radiographic bone changes and mean changes in the expressions of salivary of gingival crevicular fluid (GCF) volumes and markers^43,44^. Among the 16 included studies, eight studies contained appraisals of the outcome PPD or PD as mentioned in some prior and subsequently after the implementation of periodontal prevention or treatment modalities^12,29,32,34,38,39,41,42^. Martins et al.^29^ constitutes the only study among these that does not incorporate additional periodontal clinical parameters in association with PPD or PD^29^. Diverse plaque and gingival bleeding indices, calculus indices, CAL, frequencies of presence of pathological periodontal pockets as assessed by BoP and presence of periodontopathogens and incidences of pathological bone loss are stated parameters. Eight studies involved appraisals concerning secondary outcomes, that is evaluations of various plaque and gingival indices, alterations in the expressions of salivary or gingival crevicular fluid markers and volumes^28,30,31,33,35–37,40^. Tanaka et al.^42^ observed significant enhancements regarding CAL within diseased sites following non-surgical periodontal treatment but did not conclude gain in CAL^42^. Zaldivar-Chiapa et al.^41^ observerd statistically significant enhancements and gain in CAL with both non-surgical periodontal treatment and surgical modalities within periodontal pockets exceeding 4 mm. No statistically significant increase in CAL where observed in pockets under 4 mm. The corresponding study observed as well statistically significant improvements considering PPD or PD reductions in 1–3 mm deep pockets following non-surgical periodontal treatment in comparison with surgical modalities^41^. Alveolar bone loss or radiographic bone loss was solely involved in two studies, in which one study observed that the mean radiographic bone loss was significantly greater in DS subjects compared to systematically healthy individuals and the other study observed that the frequency of the incidence of pathological bone loss was significantly larger within DS individuals with irregular periodontal treatment compared to individuals with regular treatments^38,39^. Several studies utilized diverse configurations and concentrations of chlorhexidine, in which significant enhancements could be observed and decreased scores in periodontal clinical parameters such as PI, GI and BoP^28,37,40^. PPD was significantly improved as well when chlorhexidine was utilized in conjunction with non-surgical or surgical periodontal treatment^41^. Freedman et al.^32^ implicated few statistically significant alteration on periodontal clinical parameters when various configurations, concentrations and frequencies of applications of chlorhexidine were implemented. The corresponding study concludes that daily application of chlorhexidine with the concentration of 1%, in combination with a 6-monthly prophylaxis, may exhibit an sufficient amount of efficacy at preserving periodontal health in individuals with DS^32^. Cichon et al.^12^ constituted the solely study in which periodontal clinical parameters persisted unaltered prior and subsequently after the implementation of oral hygiene instruction and professional tooth cleaning. The prophylactic measures and tooth cleaning were conducted though at baseline and with the lack of assistance throughout the experimental period^12^.

### Quantitative synthesis

Four studies were ultimately included in the quantitative syntheses after the assessments of the heterogeneity, the variability in treatment procedures, populations and settings across studies.

**Fig. 2:**
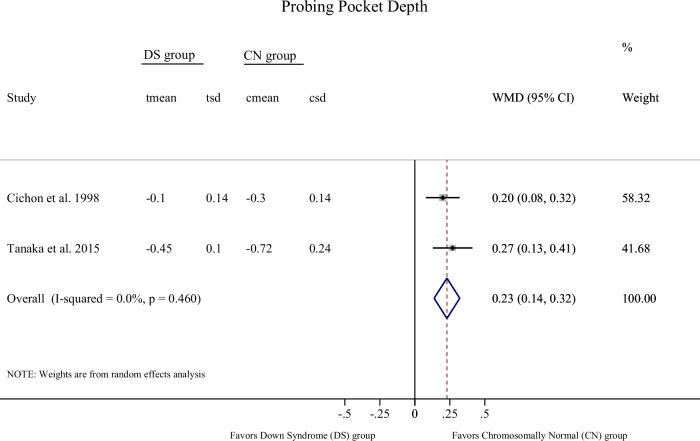
Forest plot on mean change in Pocket Probing Depth (PPD) in Down Syndrome (DS) versus Chromosomally Normal (CN) subjects receiving Professional tooth cleaning program (scaling and polishing) and Oral Hygiene Instructions for a 4–6 weeks period. The forest plot illustrates the weighted mean differences (WMD) with 95 % confidence intervals (CI) for included studies as well as the overall effect sizes.

**Fig. 3:**
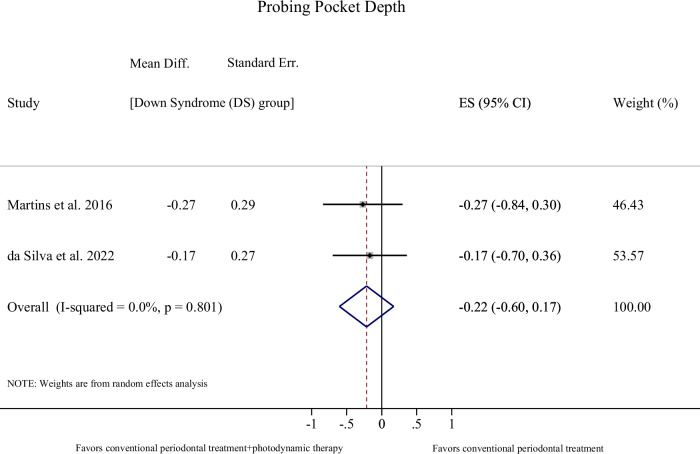
Forest plot on mean change in Pocket Probing Depth (PPD) in Down Syndrome (DS) patients receiving conventional periodontal treatment (root scaling- polishing) supplemented by photodynamic therapy versus conventional periodontal treatment alone, for a 4 weeks period. The forest plot illustrates the weighted mean difference (WMD) with 95 % confidence intervals (CI) for included studies as well as the overall effect sizes.

As per the outcome PPD, we identified evidence of a statistically significant difference in reduction in PPD after the implementation of a professional tooth cleaning program supplemented by oral hygiene instructions for 4–6 weeks, in favor of chromosomally normal (CN) subjects, compared to DS patients [2 studies, Mean difference (MD): 0.23; 95% Confidence Interval (CI): 0.14, 0.32; *p*-value < 0.001; I^2^: 0.0%; Table 1, Fig. 2]. The additional use of photodynamic therapy to conventional periodontal procedures (root scaling and polishing) in DS subjects did not appear to influence PPD change in a 4-week period [2 studies, MD: −0.22; 95% CI: −0.60, 0.17; *p*-value: 0.27; I^2^: 0.0%; Table 1; Fig. 3].

**Table 1:** Results of quantitative syntheses (meta-analyses) across primary and secondary outcomes for the eligible comparisons [all changes represent final versus baseline measurement].

Comparison [population]	Outcome	Effect size [mean difference]	Confidence Interval [95% CI]	*p*-value	I-squared [I^2^] (%)	Tau- squared [τ^2^]
	**Primary**					
Professional tooth cleaning program (scaling and polishing) and Oral Hygiene Instructions **[DS versus CN**]^f^	Change in PPD^a^ [4–6 weeks]	0.23^d^	0.14, 0.32	**<0.001**	0.0	0.0
**Conventional periodontal treatment supplemented by photodynamic therapy versus Conventional periodontal treatment** [DS only]^g^	Change in PPD^a^ [4 weeks]	−0.22^e^	−0.60, 0,17	0.27	0.0	0.0
	**Secondary**					
Professional tooth cleaning program (scaling and polishing) and Oral Hygiene Instructions **[DS versus CN**]^f^	Change in CAL^b^ [4–6 weeks]	0.14^d^	−0.16, 0.45	0.36	70.6	0.03
Professional tooth cleaning program (scaling and polishing) and Oral Hygiene Instructions **[DS versus CN**]^f^	Change in BoP (%)^c^ [4–6 weeks]	21.1^d^	−11.3, 53.4	0.20	92.8	505.1

*DS* Down Syndrome, *CN* Chromosomally Normal, *PPD* pocket probing depth, *CAL* clinical attachment level, *BoP* bleeding on probing.

Bold values indicate statistical significance *p* < 0.001.

^a^Pocket Probing Depth.

^b^Clinical Attachment Level.

^c^Bleeding on Probing.

^d^Indicates greater reduction in the CN group.

^e^The minus (−) sign indicates greater reduction in the intervention supplemented by the photodynamic therapy.

^f^Cichon et al.^12^ and Tanaka et al.^42^.

^g^Martins et al.^29^ and da Silva et al.^34^.

The results of the remaining outcomes indicated significant heterogeneity, ranging from 70.6% to 92.8% for I-squared in the two eligible outcomes examined. There was no evidence that professional tooth cleaning program supplemented by oral hygiene instructions for 4–6 weeks, presented a significantly different effect in DS compared to CN subjects, either for change in CAL [2 studies, MD: 0.14; 95% CI: −0.16, 0.45; *p*-value: 0.36; I^2^: 70.6%; Table 1], or for change in % BoP [2 studies, MD: 21.1; 95% CI: −11.3, 53.4; *p*-value: 0.20; I^2^: 92.8%; Table 1].

Publication bias could not be ultimately explored due to the paucity of included studies in the quantitative synthesis per outcome.

### Risk of bias in the included studies

**Fig. 4:**
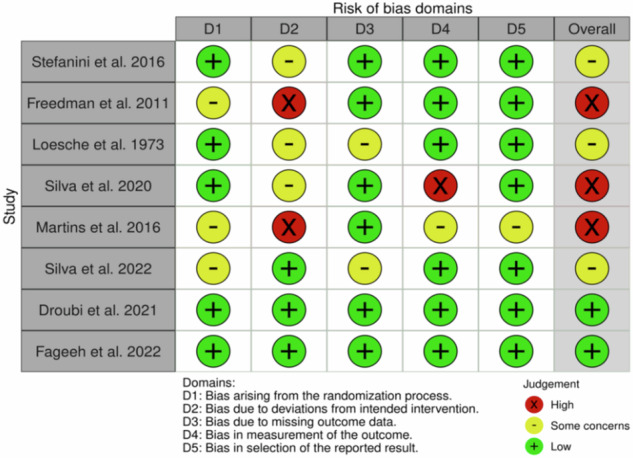
Quality assessments and risk of bias assessments of included studies. The figure summarizes the appraisals of the quality of risk of bias for included studies, accentuating fundamental domains in which potential sources of bias may impact the reliability and validity of study findings.

**Fig. 5:**
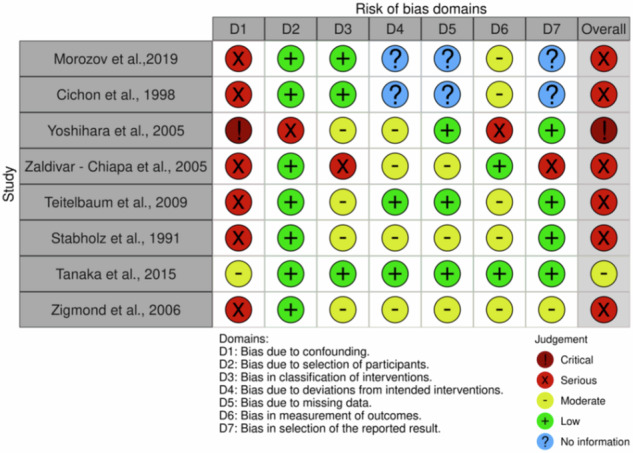
Quality assessments and risk of bias assessments of included studies. The figure summarizes the quality assessments and risk of bias appraisals for included studies, emphasizing fundamental domains in which potential bias may impact the reliability and validity of study findings.

Among the eight RCTs included, three manifested a high risk of bias in concordance with the RoB 2 guidelines^28,30,32^. Three evoked some concerns regarding their susceptibility to bias^29,31,35^. The quality assessments and risk of bias appraisals are displayed in Fig. 4. Among the eight included non-randomized studies, seven manifested substantial propensities for serious risks of bias whereas one exhibited a critical risk for bias^12,36–41^. The remaining study showed a moderate risk of bias^42^. The quality assessments and risk of bias appraisals are exhibited in Fig. 5.

## Discussion

Individuals with DS face physical and intellectual disabilities that present unique challenges across various aspects of health care, including periodontal care. This study evaluated the existing literature to determine the effectiveness of conventional periodontal care modalities in individuals with DS. The main findings of this systematic review and meta-analysis indicate that professional tooth cleaning combined with oral hygiene instructions is less effective in reducing pocket depths in individuals with DS compared to those without DS. This finding highlights the vulnerability of the DS population to progressive periodontal diseases and underscores the need for increased awareness, enhanced monitoring, preventive measures, and individualized treatment planning in agreement with previous studies^45,46^.

Non-surgical periodontal therapy, consisting of SRP, has been evidenced to efficaciously reduce PPD, notably in shallower pockets (1–3 mm), as demonstrated by Zaldivar-Chiapa et al.^41^, implicating SRP as a more favorable treatment modality in periodontal disease manifestation of early stages. The finding may contribute to ameliorated periodontal health without the requirement of invasive treatment modalities. SRP may as well be included as a frequent maintenance strategy, due to the non-invasive character, so as to preclude periodontal disease progression. Furthermore, SRP may potentially contribute to higher compliance levels in DS individuals since DS individuals may experience amplified anxiousness regarding the implementation of surgical treatment modalities. Surgical flap debridement, in contrast, incorporates more comprehensive interventions for PPD reduction, notably in PPD exceeding 4 mm^41^. Even though PPD reductions were not statistically significant in Zaldivar-Chiapa et al.^41^, surgical flap debridement has demonstrated substantial gains in CAL and significant PPD reductions as evidenced by Heitz-Mayfield et al.^47^. These findings indicate that surgical flap debridement may be more adequate in more severe manifestations of periodontal disease, although the requirement for post-operative care heightens due to remediation of long duration.

Freedman et al.^32^ and Morozov et al.^36^ revealed that frequent implementation of oral hygiene regimens and periodontal support treatments at follow-ups critically enhanced favorable long-term periodontal health outcomes^32,36^. Cichon et al.^12^ constituted the sole study in which periodontal clinical parameters persisted unaltered prior and subsequently after the implementation of oral hygiene instructions and professional tooth cleaning^12^. The obtained outcome indicate that oral hygiene instructions and sole professional tooth cleaning may be inadequate to alter substantially periodontal clinical parameters without adjunctive, supplementary or additional invasive treatment modalities. Teitelbaum et al.^37^ demonstrated that the utilization of plaque-disclosure agents manifested in more pronounced reductions in PI in comparison to fluoridated dentifrices in adjunction with chlorhexidine. The outcome indicate that the plaque-disclosure agent contributes to more perceivable plaque accumulations, assisting individuals with DS, dependents and caregivers in terms of cleaning inaccessible regions^37^. The outcomes are in agreement with the studies of Sarnat et al.^48^, Kavvadia et al.^49^ and Shyama et al.^19^, implying that individuals with intellectual impairments as ASD and DS exhibit difficulties with oral hygiene and maintain an adequate oral hygiene due to predicaments in acquiring the dexterousness needed for adequate selfcare as well as deficient consciousness of the importance of an optimal oral hygiene^19,48,49^. The findings emphasize the requirement for individualized treatment modalities and to aid these patients sustain oral health in acceptable levels. Fageeh et al.^33^ demonstrated that toothbrushes, conformed to the requirements of individuals with DS, significantly reduce plaque accumulations and diminish bleeding indices over at a time period of 4 weeks^33^. Droubi et al.^35^ supported the obtained outcome by reporting that toothbrushes with modified handles may provide additional benefits in diminishing the accumulations of plaque and promoting gingival health, highlighting the pragmatic benefits for individuals with DS^35^.

Stabholz et al.^40^ and Teitelbaum et al.^37^ showed that application of chlorhexidine led to a greater decrease in gingival bleeding and plaque scores^37,40^. This improvement implies the effectiveness of chlorhexidine in managing periodontal health in DS patients.

Freedman et al.^32^ concluded that daily application of chlorhexidine with the concentration of 1 percent, in conjunction with a 6-monthly prophylaxis, may exhibit an sufficient amount of efficacy at preserving periodontal health in DS individuals^32^. Despite exhibited improvements, the variety of concentrations, configurations and application frequencies of chlorhexidine have generated fluctuating and inconsistent outcomes in individuals with DS. This diverseness indicates the necessity of standardized treatment protocols in order to accomplish more consistent treatment outcomes. The inconclusiveness and variability in obtained outcomes emphasizes the paucity of information regarding the favorable usage of chlorhexidine, which are in accordance with previous research, indicating the requirement for elaborated and extensive studies to determine percipient guidelines^8^. Martins et al.^29^ analyzed the effectiveness of SRP alone and in conjunction with aPDT regarding the reduction of PPD. The outcomes demonstrated that SRP in conjunction with aPDT did not exhibit statistically significant discrepancies regarding the PPD reduction in comparison to SRP alone^29^. These findings are in accordance with the outcomes of Silva et al.^34^. In order to comprehend the advantage and long-term effects of aPDT and treatment efficacies, further research is deemed necessary, involving sample sizes, of greater magnitude and follow-up monitoring of long duration.

Zigmond et al.^39^ and Tanaka et al.^42^ incorporated microbiological analysis in addition to conventional mechanical periodontal treatment, providing a more profound comprehension of the periodontal health status in the DS population. Tanaka et al.^42^ demonstrated that the periodontopathogen bacterial counts in diseased sites of DS subjects did not significantly reduce following non-surgical periodontal therapy contrary to healthy controls. The obtained findings indicate that DS individuals may display inherent unfavorable microbiological responses to standard periodontal prevention and treatment modalities, emphasizing the requirement for individualized periodontal treatment protocols for DS patients, in order to manage periodontopathogens and bacterial counts more efficiently. Discrepancies within the microbiological flora, structure and levels of expressions between DS and systematically healthy individuals suggest inherent diverseness within the subgingival microbiota, which might contribute to enhanced susceptibility and periodontal disease severity in DS individuals^39,42^. Sakellari et al.^16^ demonstrated significantly higher periodontal inflammation indices clinically, and early colonization of periodontopathogens in comparison to age-matched systematically healthy and CP individuals^16^. The findings imply that DS individuals demonstrate a predisposition for a grievous periodontal disease profile from an early age. Knocht et al.^50^ exhibited similarly that DS individuals manifested more extensive CAL and greater concentrations of periodontopathogens, emphasizing that a predisposition for a more severe manifestation of periodontal disease prevails^50^. The findings are in agreement with previous research that implicates alterations in periodontopathogens within the subgingival microbiota and alterations in inflammatory mediators in DS individuals, implicating an underlying immunological or microbiological foundation for the intensified periodontal disease manifestation^13,14,16^. The inclusion and integration of microbiological analysis in periodontal research by Zigmond et al.^39^ and Tanaka et al.^42^ establishes a base for future research as it demonstrates the significance of comprehending immunological and microbiological profiles in order to construct efficacious individualized treatment protocols.

Despite the rigorous methodology employed for study selection and data extraction, the systematic review and meta-analysis has limitations. The study designs constituted randomized clinical trials, clinical trials, prospective longitudinal studies and retrospective studies, studies with alternating research methodologies as well as varieties in periodontal prevention and treatment modalities, indicating heterogeneity which may influence the equivalence and generalizability of obtained outcomes. The significant heterogeneity indicates substantial variability among individual studies. The combined effect size may therefore not meticulously reflect true effects of implemented modalities, contributing to predicaments in the interpretation of pooled outcomes with certainty. The generalizability across diverse populations and settings might be low, indicating that treatment effects alter significantly across studies. Meta-regression or subgroup investigations could aid in distinguishing variability sources and comprehending the influences of various factors in relation to the outcomes in order to manage the high heterogeneity, however this cannot be done before more clinical studies emerge in the relevant literature domain. Additionally, limited sample sizes and lack of long-term reassessments may hinder the accurate calculation of necessary statistical assumptions to detect potential significant differences. Moreover, the disparities concerning the age range of the individuals included may impact the obtained outcomes due to the variability within the developmental stages of the subjects. In addition, a substantial amount of studies exhibited serious risk of bias due to confounders. The high risk of bias of included studies may influence the internal validity of obtained outcomes, implying that noted effects may potentially be due to biases rather than substantive effects of implemented modalities. Moreover, the actual effect size may be over or undervalued, which could contribute to erroneous pooled estimates in the meta-analysis. Up to date, no previous systematic review with meta-analysis has been reported determining the treatment efficacy of periodontal prevention programs and treatment modalities in individuals with DS. The statistically significant difference in reducing PPD following a professional tooth cleaning program combined with oral hygiene instructions among DS subjects compared to CN provides newer insights. Two studies with PPD as outcome displayed low heterogeneity and were included in the meta-analysis. As the studies were conducted across eleven countries, it is evident that there is a global effort to maintain the periodontal health status of individuals with DS, underscoring the significance of future research within the discipline.

## Conclusion

Based on the best available evidence, professional tooth cleaning combined with oral hygiene instructions appears to be less effective in reducing pocket depths in individuals with DS compared to those without DS. The presence of moderate to high risk of bias in the included studies underscores the need for rigorously designed studies that minimize bias through effective blinding, randomization, control of confounding factors, and diverse treatment outcomes to further investigate these associations. Tailored periodontal care strategies that address the specific needs of individuals with DS should be implemented to improve treatment outcomes for this population.

## Supplementary information

Appendix 1

SI Table 1

Prisma2020 Checklist

## Data Availability

The authors confirm that the data supporting the findings within this study are available within this article.
