# Supplementary material for: Efficacy of periodontal treatment modalities in Down syndrome patients: a systematic review and meta-analysis
Source: Evid Based Dent. 2024 Aug 25;25(4):213–4. doi: 10.1038/s41432-024-01055-x (PMC11661967; doi:10.1038/s41432-024-01055-x)
Supplement: Supplementary file 1 — Appendix 1 [file 41432_2024_1055_MOESM1_ESM.pdf]

("Intellectual disability" OR "Chromosome Disorders" OR "Down syndrome" OR "Downs syndrome" OR "Down's syndrome" OR "Down disease" OR "Downs disease" OR "Down's disease" OR "Mongolian idiocy" OR "Mongolism" OR "Trisomy 21" OR "Partial trisomy 21" OR "Trisomy G" OR "Trisomy 21, meiotic nondisjunction" OR "Trisomy 21, mitotic nondisjunction" OR "47,xx+21" OR "47,xy+21" OR "Translocation 15 21 22" OR "Trisomy 21 syndrome") AND ("Periodont*" OR "Peridont*" OR "Parodont*" OR "Paradont*" OR "Paradent*" OR "Pericement*" OR "Paraodontopathy" OR "Pyorrhea Alveolaris" OR "Pericement*" OR "Alveolodental Osteoperiostitis" OR "Cementoperiostitis" OR "Gingivit*" OR "Gingivos*" OR "Gingival Inflammation" OR "Gingival Inflammation" OR "Epulis*" OR "Fusospirillosis" OR "Phagedenic Gingivitis" OR "Ulcerative Stomatitis" OR "Vincent*" OR "Trench Mouth" OR "Giant Cell Granuloma" OR "Peripheral Giant Cell Granuloma" OR "Peri-Implant*" OR "Apical Alveolar Abscess*" OR "Apical Dentoalveolar Abscess*" OR "Dental Granuloma*" OR "Atrophy Of Periodontium" OR "Lateral Abscess*" OR "Lateral Alveolar Abscess*" OR "Parietal Abscess*" OR "Dentin Granuloma*" OR "Tooth Granuloma*" OR "Tooth Furcation Defect*" OR "Gingival index" OR "Plaque index" OR "Oral hygiene" OR "Oral microbiome")
