# Supplementary material for: Efficacy of periodontal treatment modalities in Down syndrome patients: a systematic review and meta-analysis
Source: Evid Based Dent. 2024 Aug 25;25(4):213–4. doi: 10.1038/s41432-024-01055-x (PMC11661967; doi:10.1038/s41432-024-01055-x)
Supplement: Supplementary file 2 — SI Table 1 [file 41432_2024_1055_MOESM2_ESM.pdf]

SI Table 1. Study characteristics of included studies.

Author, year, country	Study design, timeframe	Subjects, sex	Age	Periodontal parameters	Periodontal prevention, treatment modalities	Main results
Stabholz et al., 1991, Israel	Prospective longitudinal study, 3 weeks	30 institutionalized DS patients	8 – 13 years	PII, GI, Number of papillae that bled upon a gentle insertion of wooden toothpick interproximally, index teeth (16, 12, 11, 21, 22, 26, 46, 42, 41, 31, 32, 36)	Group I (n = 10): Coating with ethyl cellulose solution containing chlorhexidine (CHX) Group II (n = 10): Coating with placebo solution Group III (n = 10): No application of substance Application every 3 days for 21 days	Group I (n = 10) – Reduction in PII, GI and number of papillae that bled Similar clinical improvements to group II with no statistical differences compared Group I and II – reductions in periodontal parameters with statistical differences (PII, GI) compared to group III
Teitelbaum et al., 2009, Brazil	Cross – over clinical trial, experimental period 10 days with a 15 day washout	40 institutionalized DS patients	7 – 13 years	Marginal gingival bleeding, PI, index teeth (11, 31, 16, 26, 36, 46)	Group I (n = 10): Fluoridated dentifrice Group II (n = 10): Fluoridated dentifrice + CHX Group III (n = 10): Fluoridated dentifrice + CHX + plaque – disclosing agent Group IV (n = 10): Fluoridated dentifrice + plaque disclosing agent Experimental period 10 days, 15 day washout Instructions on oral hygiene + orientation for parents and patients	Intra – group comparison, significant differences between PI initial and final in all groups ($p < 0.001$) Group I: 15 %, group II: 11 %, group III: 64 %, group IV: 65 % Intra – group comparison, significant differences between GI initial and final in all groups ($p < 0.001$) Group I: 8 %, group II: 21 %, group III, 37 %, group IV: 18 % Significant differences (PI reduction) between groups I and II ($p < 0.001$) Significant differences (PI reduction) between groups III and IV ($p < 0.001$)
Freedman et al., 2011, Ireland	Cross – over randomized trial, 24 months	27 DS patients, 12 males and 15 females	9.2 – 43.1 years Mean age: 25.4 years	Modified gingival index, gingival bleeding index, calculus index, plaque index,	Phase I: 1 % CHX varnish applied 3 – monthly + 3 monthly professional prophylaxis + 1 %	There were significantly lower mean pocket probing depths ($p = 0.026$) and modified gingival indices ($p = 0.029$) for the control phase compared to phase I.

				PPD at index sites (16, 11, 26, 36, 41, 46)	CHX gel applied at home daily<br>Phase II: 40 % CHX varnish applied 6 – monthly + 6 – monthly professional prophylaxis + 1 % CHX gel applied at home daily<br>Control phase: 1 % CHX gel applied at home daily + 6 – monthly professional prophylaxis<br>Experimental period 9 – 12 months, 3 months washout period<br>Patients, parents and carers instructed on oral hygiene measures + at – home topical application of CHX	There was no significant difference between phase one and the control phase with regard to gingival bleeding index, calculus index, plaque index<br>There were significantly lower mean gingival indices (p = 0.028) for phase two compared to the control phase.<br>There was no significant difference between phase two and the control phase with regard to modified gingival index, probing pocket depth, calculus index, plaque index.
Cichon et al., 1998, Germany	Controlled clinical trial, 12 weeks	10 DS patients, 6 males and 4 females<br>11 CP patients, 7 males and 4 females	DS patients: 20 – 31 years, mean age: 26.3 years<br>CP patients: 23 – 53 years, mean age: 36 years	PI, GI, PD, CAL, 4 sites per tooth/full mouth	Professional tooth cleaning program + oral hygiene instructions (Bass method of brushing) at baseline	In DS patients, mean PI, GI scores, PPD, CAL remained unchanged and showed no statistically significant change.<br>Mean percentage (%) of sites with BoP + PPD ≤ 3 mm, 4 – 6 mm, ≥ 7 mm displayed no improvement
Zaldivar – Chiapa et al., 2005, Mexico	Prospective cohort study with split – mouth design, 1 year	14 DS patients, 9 males and 5 females	17 – 30 years<br>Mean age: 25 years	PI, GI, PD, CAL, 6 sites per tooth/full mouth	Scaling and root planing.<br>Surgical flap debridement.<br>Polishing weekly for 8 weeks and oral hygiene included 0.12 % CHX rinses twice a day. Maintenance every 2 weeks for 4 months after that and once a month subsequently until completing the study.	Significant decreases in PI, GI, PD scores for both treatment methods (p < 0.001). No significant differences between treatment procedures.<br>PD for 1 – 3 mm were statistically significantly improved with non – surgical in comparison with surgical methods. Surgical treatment showed greater reduction in PD than non – surgical therapy for pockets > 4 mm although the difference were not statistically significant.<br>Significant improvement in CAL with both types of therapies in PD > 4 mm,

						however both treatments showed an increase in CAL in pockets < 4 mm.
Morozov et al., 2019, Russia	Prospective clinical trial, unspecified timeline	38 DS patients<br>64 systematically healthy patients	8 – 12 years	Patient hygiene performance index	Prevention program comprising of 3 stages:<br>Stage 1: Preparatory stage – adaption to dental clinical settings<br>Stage 2: Preventive and educative activities – instructions on oral hygiene measures + orientation<br>Stage 3: Stage of execution – implementation of preventive procedures and dental hygiene monitoring	Intra – group comparison, significant difference between PHP index initial and final in both groups ($p < 0.05$)
Yoshihara et al., 2005, Japan	Retrospective cohort study, 3.7 ±1.3 months managed group, 27.5 ± 10.1 months; interrupted group	24 DS patients, 18 males and 6 females.<br>Managed group: 13 (10 males and 3 females)<br>Interrupted group: 11 (8 males and 3 females)	14.4 – 36.8 years<br>Mean age: 20.8 ± 5.6 years	Modified total PMA, PD, frequency of the presence of a pathological periodontal pocket, ABL, frequency of the incidence of pathological bone loss	Professional tooth cleaning, various combinations of scaling and counseling for caregivers regarding periodontal disease	PD and frequency of a pathological periodontal pocket were significantly higher in the interrupted group than in the managed group ($p < 0.001$)<br>ABL and frequency of the incidence of pathological bone loss in the interrupted group were significantly larger than those observed in the managed group ($p = 0.031$)<br>M - PMA in the interrupted group was significantly higher than that in the managed group ($p = 0.045$)
Stefanini et al., 2016, Italy	Randomized controlled clinical trial, 2 weeks	56 DS patients<br>Test group: 28 (9 males, 19 females)<br>Control group: 28 (12 males, 16 females)	6 – 18 years<br>Mean age test group: 9.6 years<br>Mean age control group: 10.6 years	PI, 4 sites per tooth/full mouth	Oral hygiene instructions + usage of either Digital Brush (TNT gauze sanitizing and antiseptic soaked with CHX 0.12 %) or normal sterile gauze soaked in water	Plaque index improvement from T0 – T1 in control group was 11.7 %, showing a significant difference from T0 ($p < 0.001$)<br>Plaque index improvement from T0 – T1 in test group was 24.1 %, showing a significant difference from T0 ($p < 0.001$)<br>Mean difference between test and control group were statistically significant ($p < 0.001$)

Martins et al., 2016, Brazil	Controlled, randomized, split–mouth clinical trial, 1 month	13 DS patients	23 – 46 years<br>Mean age: 33.9 years	PPD, 6 sites per tooth/full mouth	SRP or SRP + aPDT<br>Oral hygiene instructions	Periodontal conditions were improved among all the participants.<br>Non – significant reduction in PPD in the aPDT – with SRP group relative to that of the SRP group.<br>A significant reduction in median PPD was observed in both groups ($p < 0.001$)
Silva et al., 2020, Brazil	Randomized, cross – over clinical trial	29 DS patients (11 males and 18 females)	6 – 14 years<br>Mean age: 9.03 years	Quigley Hein Index	7 – day manual or electronic toothbrush period. 23 – 25 hours without oral hygiene procedures. 7 – day washout period and subsequently cross over.	Significant decrease of biofilm after brushing ($p < 0.001$), but there was no difference in biofilm before ($p = 0.390$) or after ($p = 0.985$) toothbrushing between toothbrush type.<br>Use of electronic toothbrush resulted in a decrease in biofilm greater than 70 % in the majority of participants, with no significant difference compared with a manual toothbrush ($p = 0.762$)<br>% biofilm reduction of both brushes remained at approximately 70 % regardless of age group (6 – 9 years $p = 0.919$; 10 – 14 years $p = 0.671$)
Tanaka et al., 2015, Brazil	Prospective case control (pilot) study	35 patients<br>Test group: 23 DS patients<br>Control group: 12 systematically healthy individuals	DS: 31.91 ± 5.85 years<br>Control: 41.25 ± 6.17 years ($p = 0.001$)<br>Plaque and gingival bleeding more prevalent in DS patients than in control group ($p < 0.0001$, $p = 0.0090$)	PPD, CAL, BoP, 6 sites per tooth/full mouth	Non – surgical periodontal treatment (Oral hygiene instructions + SRP) + professional plaque control program (supragingival plaque removal + reinstruction of oral hygiene procedures for patients and family/caretakers) for 45 days	Non – surgical therapy resulted in significant improvement in clinical parameters of diseased sites for both group at 45 days ($p < 0.05$).<br>No differences were observed in clinical parameters in healthy sites between baseline and 45 days after treatment for both groups.<br>No statistically significant difference between groups in counts of P. Gingivalis and T. Forsythia in diseased sites at baseline.<br>Significantly higher levels of T. Denticola in DS group than in control group ($p = 0.33$).<br>Levels of P. Gingivalis, T. Forsythia, T. Denticola significantly higher in DS group than in control group after 45 days ($p < 0.0001$, $p = 0.0003$, $p = 0.0007$).

						Levels of P. Gingivalis were significantly higher in DS group than in control group in healthy sites at baseline (p = 0.0201) but no statistically significant differences were observed in the counts of T. Denticola and T. Forsythia.<br>Forty-five days after peri- odontal treatment, for both diseased and healthy sites, the levels of P. gingivalis, T. forsythia, and T. denticola were significantly higher in the DS group than in the control group (for diseased sites: p < 0.0001, p = 0.0003, p = 0.0003, p = 0.0007; for healthy sites: p < 0.0001, p = 0.0043, p < 0.0001<br>The intragroup comparisons (between the study periods)<br>revealed that the non-surgical periodontal therapy did not significantly reduce the bacterial counts of the three investi- gated microorganisms in the diseased sites of the DS group (Wilcoxon test; p>0.05; Table 3). Otherwise, for the control group, these levels were significantly reduced 45 days after periodontal treatment. After the experimental period, the healthy sites of both groups exhibited significant reductions of all the tested microorganisms (p < 0.05)
Zigmond et al., 2006, Israel	Retrospective cohort study, 10 years	58 patients in total Test group: 30 DS patients, 17 males and 13 females. Control group: 28 systematically healthy, 9 males and 19 females	DS patients: Mean age 23.3 ± 4 years Systematically healthy:	Presence/absence of plaque at 4 sites per index tooth, BoP, PPD, CAL at 6 sites per index tooth, radiographic bone loss	Preventive dental health program (oral hygiene instructions given to parents/guardians + supra/subgingival scaling every 6 months)	Mean plaque scores were similar in DS and control groups (55.5 % vs. 50.1 %, p = 0.6<br>Mean BoP of DS group 1.5 x greater than of the control group but did not reach statistical significance (p = 0.6)<br>Mean PPD, CAL, percentages of periodontally affected patients, sites

			Mean age: 22.8 ± 5 years			and teeth were significantly higher in DS compared with control group. Mean radiographic alveolar bone loss was significantly greater in DS subjects compared with controls. Percentages of periodontally affected patients, sites and teeth, as calculated from radiographs, were far greater in DS subjects than in controls.
Loesche et al., 1973, USA	Randomized clinical trial	15 institutionalized DS patients, 13 males and 2 females	7 – 14 years	Modified plaque and gingivitis indices at index teeth	Oral prophylaxis including some subgingival scaling at baseline + topical kanamycin sulphate paste, once or twice a day for 3 – 5 days every fifth week over 52 weeks. Teeth were not brushed during this 52 week period.	Wet weight of supragingival plaque in the Kanamycin group was significantly reduced when compared to supragingival plaque formed in the group receiving placebo paste. Gingivitis score of Kanamycin group did not deteriorate during the period of antibiotic administration despite absence of oral hygiene procedures.
Silva et al., 2022, Brazil	Randomized controlled split – mouth study, one month	8 DS patients, 2 males and 6 females	17 – 38 years Mean age 24.60	BoP, PPD at 6 sites per tooth/full mouth, PI at 4 sites per tooth/full mouth	Conventional periodontal therapy (scaling + root planing + oral hygiene instructions) + aPDT for two randomly chosen quadrants	No statistically significant differences were observed in the intergroup evaluation ($p > 0.05$) No statistically significant variations were observed in relation to plaque index ($p > 0.05$) and probing depth ($p > 0.05$) in the intragroup evaluation Significant reduction in bleeding index was observed for both test group ($p = 0.013$) and control group (0.015)
Droubi et al., 2021, Syria	Parallel – arm randomized clinical trial, 3 weeks	24 DS patients, 24 patients without special needs 22 males and 26 females	6 – 9 years	TMQHPI index full mouth at 4 times MGI index full mouth at 3 times	Sub group A (n = 12): Children with no special needs who used a conventional toothbrush Sub group B (n = 12): Children with no special needs who used a customized handle brush Subgroup C (n = 12): Children with DS who used a conventional toothbrush	Both subgroups of children with no special needs showed lower accumulation scores than DS subgroups at T0 (pre – brushing) $p < 0.05$ No significant difference was observed regarding the amount of plaque accumulation between the Sub B and Sub D groups at T1 (post – brushing at baseline) $p > 0.05$ Significant difference between Sub group A and C Significant difference between both subgroups of children with no special needs $p < 0.05$

					Subgroup D ($n = 12$): Children with DS who used a customized handle toothbrush	Sub group D had lower plaque accumulation scores than Sub group C<br>Significant difference between the subgroups in each main group at T2 (1 week) and T3 (3 weeks) $p < 0.05$; between Sub group A and C $p < 0.05$; and Sub group B with Sub group D $p < 0.05$<br>Each subgroup showed an improvement in plaque accumulation scores in time (T0 to T3) $p < 0.05$, except for T2 and T3 of Sub group A, which presented no significant difference $p > 0.05$<br>Significant difference at baseline between the Sub A and Sub C groups and between the Sub B and Sub D groups regarding MGI<br>Significant difference was found at T2 between the Sub A and Sub C groups and between the Sub B and Sub D groups<br>Significant difference was observed between all each paired groups at T3<br>Significant difference for each group at all studied periods, where a considerable improvement of MGI was observed
Fageeh et al., 2022, Saudi Arabia	Single – center parallel – group randomized clinical trial, 8 weeks	16 DS patients	6 – 15 years	PI, BI of index teeth (16, 21, 24, 36, 41, 44)	Conventional toothbrush given with instructions for use (Fone's method) at baseline (T0)<br>Group I ($n = 8$): Allocated to Curved Brush 4 weeks later (T1)<br>Group II ($n = 8$): Allocated to Superfine Nano Brush 4 weeks later (T1)	Statistically significant reductions were noted between visits in both groups ($p < 0.05$)<br>No notable changes in mean plaque index were observed between the two groups at each visit ($p > 0.05$)<br>Statistically significant reductions were noted between visits in both groups ($p < 0.05$)<br>No statistically significant changes in the bleeding index were seen between the two groups at each visit ($p > 0.05$)<br>Both indices showed statistically significant improvement with the use of special needs toothbrushes at T2 (8 weeks after T0) compared to the regular brushes at T1 ($p < 0.05$)

No statistically significant difference was observed between the two special needs toothbrushes with regards to CFU counts ($p > 0.05$)
